# Osteosarcoma immune microenvironment: cellular struggle and novel therapeutic insights

**DOI:** 10.3389/fimmu.2025.1584450

**Published:** 2025-06-04

**Authors:** Yi Zhang, Shasha Jiang, Jing Lv, Wei Feng, Yan Yu, Heping Zhao

**Affiliations:** Department of Clinical Laboratory, Honghui Hospital, Xi’an Jiaotong University, Xi’an, China

**Keywords:** immune cell, tumor microenvironment, osteosarcoma, tumor-associated macrophages, immunotherapy

## Abstract

Recent advances in immunotherapy have shown remarkable success across multiple solid tumor types, revitalizing interest in immune-modulating strategies for osteosarcoma (OS). Within the complex tumor microenvironment (TME), immune cells exhibit dual regulatory roles-exerting critical regulatory influences on both tumorigenesis and disease progression while simultaneously serving as therapeutic targets. Particularly in OS, the dynamic interplay between malignant cells and the unique bone microenvironment manifests through intricate immune cell-mediated crosstalk. This comprehensive review analyzes the paradoxical roles of immune cell subsets in OS pathophysiology, detailing their tumor-promoting versus tumor-suppressing functions. Furthermore, we systematically evaluate recent progress in immune cell-targeted therapeutic approaches, including adoptive cell therapies and macrophage reprogramming strategies. The review encompasses current clinical applications and emerging preclinical innovations, providing valuable insights for optimizing immunotherapeutic approaches in OS management.

## 1 Introduction

Osteosarcoma (OS) is the most common primary malignant bone tumor, commonly found in children and adolescents with a median age of 16 years (1, 2). The overall age-standardized incidence rate of OS was 5.2 cases per million (3). It ranks eighth among childhood cancers and accounts for merely 2% of all cancers in children, adolescents, and young adults (4, 5).The incidence of OS varies based on age, race, gender, and other factors, exhibiting a bimodal distribution with peak rates in the 10–19 and 60–79 age groups (6).

OS is characterized by malignant osteoblasts capable of producing immature bone or osteoid tissue. It typically arises in long bones near the epiphyseal growth plate, with the femur (52%), tibia (24%), and humerus (10%) being the most common sites (7, 8). OS exhibits significant heterogeneity at both histological and genetic levels. Histologically, it can be classified into various subtypes based on differentiation components. Genetically, OS demonstrates extensive intratumoral heterogeneity, including somatic copy number alterations, structural variations, and a limited number of gene mutations (9). Advances in next-generation sequencing have enabled comprehensive genomic mapping of OS, revealing that nearly every case harbors genomic alterations, suggesting that the heterogeneity of OS may exceed our current comprehension (10). Stratifying patients based on genetic characteristics is expected to facilitate more effective targeted treatments, advancing the era of precision medicine (10). The majority of OS cases exhibit genomic alterations in TP53, particularly TP53 and RB inactivation (11). However, the precise etiology remains elusive, and investigations into pathogenesis have uncovered various potential pathways, with chromosomal instability being the most widely accepted mechanism driving tumor development through chromosome trisomies or clusters (5).

Currently, the 5-year survival rate for patients with localized OS is approximately 60%, dropping to 20% in cases of recurrence or metastasis (12). Prior to the 1970s, patients relying solely on surgery had a 5-year overall survival rate of around 20%. However, the integration of surgical resection with multidrug chemotherapy has increased survival rates to 60-70%. For patients with recurrent or unresectable OS, the prognosis remains particularly poor, with a 4-month event-free survival (EFS) rate of only 12% (13). For resectable recurrent disease, such as typical lung metastases, the median EFS is 4 months, with a 2-year EFS of 12% (14). Over the past decade, there has been some improvement (3), but compared to advancements in other solid tumor treatments, OS prognosis has not significantly improved (15, 16). The reasons for this disparity are multifaceted, including an inadequate understanding of OS genetic complexity (16), unclear mechanisms related to prognosis, and challenges in accurately assessing individual patient prognosis due to tumor heterogeneity (17, 18). Additionally, OS patients often exhibit resistance to conventional chemotherapy, and high-dose chemotherapy can lead to severe adverse effects, including neutropenia, infectious complications, and thrombocytopenia (3). Studies have shown that the tumor microenvironment (TME) appears to influence clinical outcomes and treatment response in OS (19, 20). Therefore, understanding the regulatory mechanisms of TME in OS will help provide targets for OS treatment.

## 2 Traditional therapies for OS and associated challenges

OS is the most common primary malignant bone tumor, frequently leading to lung metastasis, which is the primary cause of death among OS patients (21). Traditional treatments include complete resection of detectable tumor tissue and multi-drug chemotherapy regimens, typically comprising doxorubicin, high-dose methotrexate, cisplatin, and ifosfamide (22). However, a Phase II trial (OSAD93) found that the omission of doxorubicin in the preoperative regimen resulted in excellent long-term survival outcomes in the treatment of localized OS with limited toxicity (23). Nevertheless, preoperative (neoadjuvant) followed by postoperative (adjuvant) chemotherapy is the preferred approach, facilitating safer surgery and the preparation of appropriate prostheses (22). For recurrent and metastatic OS, resection can offer survival benefits (24). A Phase II randomized clinical trial assessed whether the addition of lenvatinib to the ifosfamide and etoposide regimen could improve outcomes in pediatric patients with OS (25). The results indicated that it did not significantly improve progression-free survival but highlighted the importance of conducting randomized clinical trials in patients with recurrent OS (25). Compared with the previously reported retrospective use of gemcitabine in combination with docetaxel for recurrent OS, the combination of gemcitabine and nab-paclitaxel demonstrated similar clinical activity and toxicity (26). Chemotherapy regimens such as ifosfamide/etoposide or gemcitabine/docetaxel show some efficacy in unresectable recurrent disease (27). Surgical resection combined with neoadjuvant and postoperative chemotherapy can elevate the long-term survival rate of localized OS patients to 70%. However, for recurrent and metastatic OS, the long-term survival rate remains below 20%, and the standard treatment strategy has remained unchanged for decades (28). Thus, there is an urgent need to develop novel treatments to enhance overall survival, particularly for recurrent andmetastatic OS.

A study has found that patients who develop surgical site infections after primary tumor resection have better outcomes (29). This suggests the potential therapeutic role of immune activation in the treatment of OS. Although there are currently few clinical trials investigating immunotherapy for OS, these studies do provide support for further research into immunotherapeutic approaches. A Phase I/II study (Phase I: NCT02173093) confirmed the safety and potential clinical benefits of T cells carrying anti-CD3×anti-GD2 bispecific antibodies in patients with recurrent/refractory disease (30). Some immune checkpoint inhibitors have also demonstrated efficacy in the treatment of OS. The results of the Phase II IMMUNOSARC study (NCT03277924), which investigated the efficacy of nivolumab in combination with sunitinib in adult patients with advanced OS, showed that over 15% of patients were progression-free at 6 months; however, 17% of patients discontinued treatment due to toxicity, highlighting the need for careful consideration of the toxicity profile of this regimen (31).An open-label Phase I/II trial verified the safety and preliminary efficacy of the anti-programmed death-ligand 1 (PD-L1) antibody as maintenance therapy in patients with locally high-risk OS (32). As our understanding of OS deepens, oncologists increasingly recognize the challenges of solely targeting OS cells to improve prognosis. Consequently, the focus is shifting towards the TME of OS (33).

## 3 The dual role of immune cells in TME

The TME is a highly heterogeneous and complex ecosystem comprising tumor cells and various non-tumor cells embedded within an altered extracellular matrix. Growing research underscores the significance of TME changes in OS tumorigenesis, proliferation, metastasis, and drug resistance, influencing patient prognosis (34–36). OS thrives within the bone microenvironment, a specialized, dynamic milieu composed of bone cells (osteoclasts, osteoblasts), stromal cells (mesenchymal stem cells, fibroblasts), vascular cells (endothelial cells, pericytes), immune cells (macrophages, lymphocytes), and a mineralized extracellular matrix (ECM). Under physiological conditions, the coordinated activities of bone, blood vessels, and stromal cells maintain bone homeostasis through robust paracrine signaling and cellular communication. The crosstalk between OS cells and the bone microenvironment involves immune cells, with the TME characterized by immune suppression, facilitating tumor immune evasion.

Immune cells in the TME play a key role in tumor occurrence and development and serve as important targets for antitumor immunotherapy (37). Low immune cell infiltration in the TME indicates weak anti-tumor immunity and aids tumor cells in evading immune attacks (38, 39). The immune microenvironment in OS comprises cellular and non-cellular components. Cellular components include immune cells such as tumor-associated macrophages (TAMs), tumor-associated neutrophils (TANs), myeloid-derived suppressor cells (MDSCs), mast cells, T cells, B cells, natural killer cells (NK cells), and dendritic cells (DCs). Non-immune cells, such as mesenchymal stem cells (MSCs) and circulating tumor cells, interact with the immune system, forming an inhibitory immune network (40).

### 3.1 Tumor-promoting immune cells

**Figure 1:**
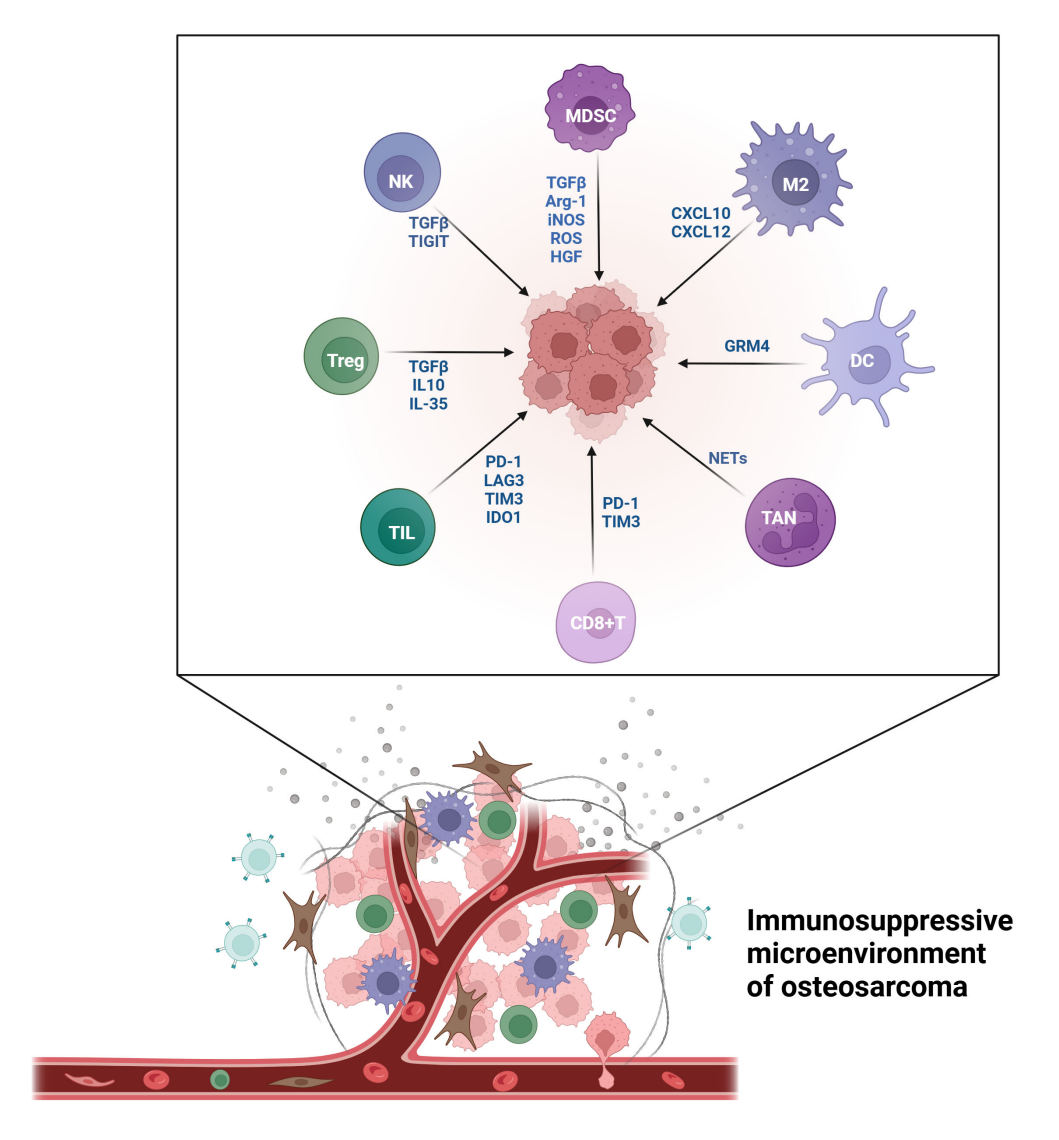
The immunosuppressive microenvironment of OS.

The TME in OS exhibits pronounced immune suppression, with immune cells displaying complex and diverse functions (**Figure 1**). Understanding the roles and mechanisms of various immune cells and developing targeted treatment strategies can enhance OS prognosis.

#### 3.1.1 Tumor associated macrophages

TAMs are the most prevalent immune cells in the TME. In OS, TAMs may constitute over 50% of immune cells, significantly influencing tumor initiation, progression, metastasis, immunosuppression, and drug resistance (33). In OS, the TME exhibits extensive macrophage infiltration, predominantly myeloid CD163^+^ cells, potentially facilitating tumor immune evasion (41). TAMs modulate local immunity, angiogenesis, and malignant cell migration, primarily promoting tumor growth by facilitating macrophage polarization towards an anti-inflammatory phenotype and enhancing immune infiltration (33).

Traditional transcriptome research, relying on mixed cell populations, lacks the resolution to identify specific cell types, failing to capture the complexity of intratumoral heterogeneity in OS. Single-cell RNA sequencing (scRNA-seq) has shown promise in investigating intratumoral heterogeneity and cell crosstalk with the TME. Yan Zhou et al. (42) identified 11 major cell clusters through RNA sequencing of 100,987 single cells from primary, recurrent, and pulmonary metastatic OS lesions. In pulmonary metastatic OS, they discovered the infiltration of pro-inflammatory FABP4^+^ macrophages. Three TAM subgroups, including M1, M2, and M3 TAMs, were identified in OS lesions, with the majority being M2 TAMs (42). M2 TAMs are recognized as the primary tumor-associated anti-inflammatory macrophages. Researches have revealed that M2-related cytokines, chemokines, and cell markers are overexpressed in pulmonary OS metastasis (43, 44). Furthermore, Shao et al. (45) demonstrated that M2 macrophages are enriched in primary OS tissue, activating tumor stem cells and inducing drug resistance. Their study found that all-trans retinoic acid (ATRA) therapy, by preventing M2 polarization of TAMs, is a promising approach for OS treatment (45).

M1 TAMs are associated with inflammatory factors. The proportion and absolute number of M1 TAMs decrease in metastatic OS (44). Interestingly, Yan Zhou et al. (42) found that M1 TAMs exhibit elevated activities in the TGF-β and Hedgehog signaling pathways, potentially inducing M2 polarization. Additionally, a small subset of M2 TAMs displays relatively high expression levels of M1 TAM marker genes, confirming the dynamic transformation between M1 and M2 TAMs within the OS TME. Minor shifts in the balance of polarized macrophages could significantly influence OS prognosis (46), and the equilibrium between M1 and M2 macrophages may play a pivotal role in OS outcomes (47). M3 TAMs, identified as FABP4^+^ TAMs, predominate in lung metastatic OS lesions. The sequencing results provide a single-cell atlas, exploring tumor heterogeneity and identifying potential therapeutic targets for OS (42). Furthermore, macrophage infiltration with an M0 phenotype in OS is correlated with poor prognosis (48). However, categorizing TAMs into M1 and M2 to analyze their effects on OS pathogenesis, metastasis, and drug resistance may be overly simplistic.

Anthony R. Cillo et al. (49) demonstrated through multiplex immunofluorescence analysis that recurrent Ewing’s sarcoma and OS exhibit increased immune infiltration compared to primary disease. ScRNA-seq analysis revealed distinct subpopulations of CD14^+^CD16^+^ macrophages in OS and identified common mechanisms underlying immune infiltration driven by these macrophages, as well as unique immune infiltration pathways facilitated by CXCL10 and CXCL12 (49).

**Figure 2:**
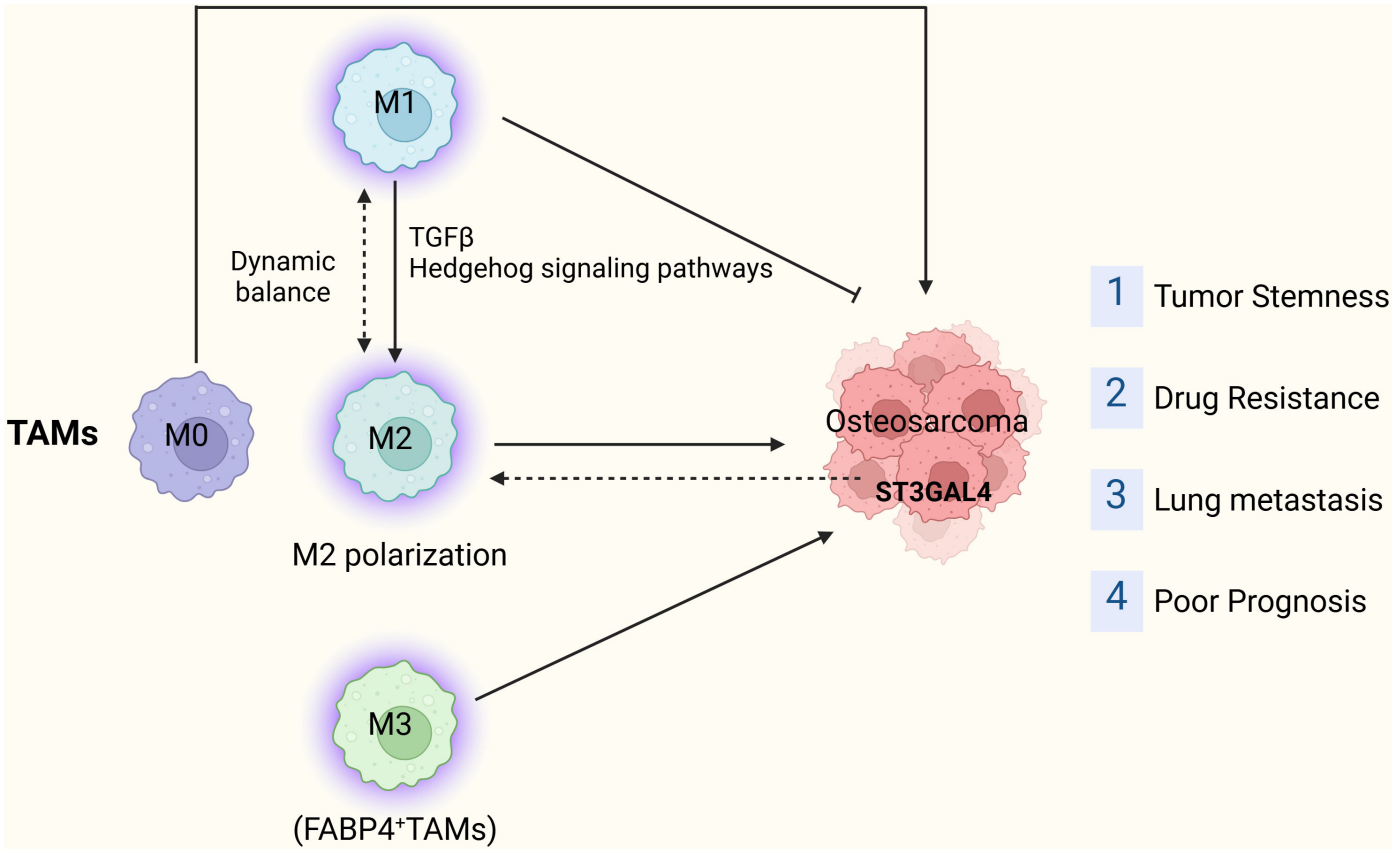
The tumor-promoting role of TAMs in OS.

Macrophage polarization plays a pivotal role in OS onset, progression, and metastasis. Consequently, modulating macrophage polarization emerges as a potential therapeutic target for OS. Certain studies have devised a risk model based on macrophage-related genes and employed molecular biology experiments to explore the function of the pivotal risk gene ST3GAL4 in OS cells. Knocking out ST3GAL4 notably suppresses OS cell proliferation, migration, invasion, and glycolysis, while also inhibiting M2 macrophage polarization (50). A recent investigation revealed that ST3GAL4 not only contributes to protein glycosylation but also influences the Siglec-7 and Siglec-9 signaling pathways by facilitating ligand synthesis in tumor cells, subsequently promoting macrophage polarization (51). These findings support considering ST3GAL4 as a promising target for tumor immunotherapy. Additionally, Jinti Lin et al. reported that MerTK-mediated efferocytosis promotes OS progression by facilitating M2 polarization and PD-L1-induced immune tolerance, modulated by the p38/STAT3 pathway (52) (**Figure 2**).

#### 3.1.2 Dendritic cells

OS exhibits infiltration of T cells and macrophages, with a significant presence of DC-SIGN/CD11c^+^ DCs within these infiltrates. In OS lesions, four distinct DC subgroups were identified: monocyte-derived CD14^+^CD163^+^ DCs, conventional myeloid-derived CD11c^+^ DCs (cDC2), CD141^+^CLEC9A^+^ DCs (cDC1), and CCR7^+^ DCs (53). CCR7 plays a role in DC chemotaxis, survival, migration speed, cellular structure, and endocytosis, all factors closely linked to tumor metastasis (53). Research has revealed that the proportion of CD11c^+^ DCs in lung metastatic lesions is 45% greater than in primary and recurrent lesions (42).

A recent Mendelian randomization analysis revealed a positive correlation between the absolute counts of CD80 and CD28^-^CD4^-^CD8^-^ T cells on CD62L^+^ myeloid DCs and OS (54). Runsang Pan et al. (55) constructed a risk model to predict OS prognosis and inform treatment strategies. Their study indicated that high infiltration of resting dendritic cells within OS tissue is linked to poor prognosis. The characteristics of resting DCs, derived from AOC3, CDK6, COL22A1, and RNASE6, may assist in predicting OS prognosis, thereby providing valuable guidance for treatment (55). Furthermore, Trang Le et al. reported that DC infiltration into the OS microenvironment is correlated with adverse clinical outcomes (48).

The role of DCs in the immune microenvironment is highly complex. Research suggests that DCs can drive OS development through oncogenes and tumor suppressor glutamate receptor subtype 4 (GRM4) (56).

#### 3.1.3 T cells

T cell infiltration plays a pivotal role in the anti-tumor immunity of OS, exhibiting significant heterogeneity in its classification. In OS, the number of T cells in metastatic lesions is notably higher than in primary and recurrent lesions (57). Evading host immune responses through various mechanisms is a hallmark of malignant tumors. T cell activation plays a pivotal role in the tumor immune response, primarily through two pathways (58): the interaction between the T cell receptor (TCR) and major histocompatibility complex (MHC) presenting antigens, and the binding of the co-stimulatory transmembrane receptor CD28 expressed on T cells to its ligands CD80/86 (58). However, immune checkpoints such as programmed cell death protein-1 (PD-1) and cytotoxic T lymphocyte-associated antigen-4 (CTLA-4) can negatively regulate or disrupt these pathways by binding to PD-L1 and CD80/86, respectively (58). These PD-1/PD-L1 or CTLA-4/CD80/86 co-inhibitory signaling pathways induce TME immune tolerance, preventing the immune system from damaging cancer cells and ultimately leading to immune evasion (57).

The concentration of tumor-infiltrating lymphocytes (TILs) expressing immune checkpoints and immune regulatory molecules, such as PD-1, PD-L1, lymphocyte activation gene 3 (LAG-3), T-cell immunoglobulin and mucin domain protein 3 (TIM-3), and indoleamine 2,3-dioxygenase (IDO1), is notably higher in lung metastases (41). The immune microenvironment within tumor lesions is inhibitory, featuring a higher number of TIM-3^+^PD-1^+^ T cells compared to those in peripheral blood. Furthermore, the specific immune suppression of TIM-3^+^PD-1^+^ T cells is amplified by M2 TAMs (59). Additionally, data indicates that TIM-3/galectin-9 (Gal-9) signaling significantly promotes the apoptosis of CD4^+^ and CD8^+^ T cells within the OS TME, resulting in poor prognosis for OS patients (60). Depleting CD163^+^ macrophages can notably enhance T cell proliferation and the production of pro-inflammatory cytokines (59). TILs are primarily distributed in areas expressing human leukocyte antigen class I, whereas CD4^+^ and CD8^+^ T cells are concentrated at the interface of lung metastases (41). Furthermore, in the first reported case of extramedullary OS characterized by the spontaneous regression of the primary lesion, T cells that are CD8-positive, T-cell restricted intracellular antigen-1 (TIA-1)-positive, and granzyme B-positive appeared to infiltrate the primary lesion, suggesting that the immune system may have played a role in triggering the spontaneous tumor regression (61). Sun et al. demonstrated that CD8^+^ T cells exhibit low infiltration in OS tissues and induce OS proliferation (62). In the OS TME, CD8^+^ T cells are dysfunctional, accompanied by increased expression of PD-1 and TIM-3. TIM-3 blockade can restore the allogeneic response function of CD8^+^ T cells both *in vivo* and *in vitro (* 62). This also implies that the exhaustion of CD8^+^ T cells in the TME inhibits anti-tumor immunity.

#### 3.1.4 Regulatory T cells

Tregs play a dual edged role in the pathogenesis of OS. Not only do they assist tumor cells in evading the body’s immune surveillance, but they also play a key part in promoting tumor angiogenesis (63). Within the microenvironment of OS, Tregs operate through various mechanisms. They secrete immunosuppressive cytokines, including IL-10, IL-35, and TGF-β, to hinder the activity of effector T cells and suppress osteoclast formation via direct cell contact-dependent means (64). The interaction between CD4^+^ Treg cells and osteoclasts significantly alters the TME and correlates with a poor prognosis in OS (64). Based on the mathematical model of OS, it was discovered that the number of Treg subsets initially decreased and then increased (65). Additionally, Yan Zhou et al. reported that Tregs expressing T cell immune receptors containing Ig and ITIM domains (TIGIT) infiltrate OS tissues, offering a novel target for immunotherapy of OS (42). The immunosuppressive molecule Gal-9 expressed on CD4^+^CD25^+^ Tregs can facilitate the development of M2 macrophages and lead to the exhaustion of CD8^+^ T cell function, potentially serving as a key mechanism disrupting T lymphocyte response to pathogens (66). Furthermore, the interaction between CD4^+^CD25^+^ Tregs expressing Gal9 and TIM-3^+^ T cells, monocytes, as well as naive CD4^+^ T cells, can result in progressive inhibition of the Th1 response (67).

#### 3.1.5 NK cells

In the OS microenvironment, NK cells are suppressed, and TGF-β expression is elevated. TGF-β plays a pivotal role in diminishing natural killer group 2D (NKG2D) mediated tumor surveillance (68). TIGIT, serving as a marker of exhaustion, is also expressed on NK cells. TIGIT inhibits the cytotoxic activity of NK cells against tumors by interacting with ligands, specifically CD155 and CD112 (69). Recently, Shengnan Yu et al. suggested that targeting immune checkpoints and/or the TGF-β pathway could potentially rejuvenate the tumor-clearing efficiency of NK cells (70). Moreover, stimulating the intrinsic tumor-killing capability of NK cells emerges as a promising strategy (70). The infiltration of NK cells in OS is associated with gender. Hao Yang et al. conducted a thorough analysis of immune infiltration within the OS microenvironment, concluding that male patients possess 71% more NK cells compared to female patients (71). The underlying mechanism is explained as TGF-β promoting angiogenesis, bone remodeling, and cell migration by suppressing the expression of the activated receptor NKG2D and decreasing the release of NK cell-killing perforin (68).

#### 3.1.6 MDSCs

MDSCs play a pivotal role within the TME of OS. MDSCs diminish anti-tumor immunity, particularly T cell activity, by secreting substances like arginase-1 (Arg-1), inducible nitric oxide synthase (iNOS), and reactive oxygen species (ROS). In turn, this aids tumor cells in evading immune surveillance (72). Furthermore, MDSCs create a pre-metastatic microenvironment in distant organs, facilitating the spread and colonization of tumor cells (73). Additionally, they secrete TGF-β and hepatocyte growth factor (HGF), which facilitate epithelial-mesenchymal transition (EMT), thereby promoting the invasion and metastasis of OS (74). OS cell-derived extracellular vesicles activate pulmonary interstitial macrophages through the secretion of chemokine CXCL2, thereby triggering the influx of gMDSCs. High levels of S100A11 expression or a high number of circulating gMDSCs are associated with the emergence of lung metastasis and poor prognosis in OS patients (75). MDSCs can inhibit T cell proliferation, diminish T cell-mediated immune responses, and promote T cell apoptosis by depleting L-arginine and generating reactive oxygen species within the microenvironment. Additionally, MDSCs suppress the functionality of NK cells and DCs (76). Furthermore, under hypoxic microenvironment stimuli, MDSCs facilitate angiogenesis and the establishment of pre-metastatic niches, which are closely linked to OS metastasis (77). Given MDSCs’ pivotal role in immunosuppression and tumor progression, they have emerged as potential targets for immunotherapy. Therapeutic strategies targeting MDSCs may aid in alleviating immunosuppression and bolstering the efficacy of anti-tumor immune responses.

#### 3.1.7 TANs

The count of neutrophils within non-metastatic tissues was higher than that in metastatic tissues (78). Most studies on neutrophils in patients with OS have focused on the neutrophil-lymphocyte ratio or circulating neutrophils (76). An increase in the neutrophil-lymphocyte ratio before treatment or surgery may be associated with poor prognosis, suggesting that the neutrophil-lymphocyte ratio could be studied as a prognostic biomarker (76). TANs constitute a heterogeneous and functionally diverse subset of neutrophils that infiltrate the TME. TANs can be polarized into either the anti-tumor N1 or pro-tumor N2 phenotype. Infiltrating neutrophils combat tumor cells by recruiting immune cells and facilitating antibody-dependent cellular cytotoxicity (70, 79). Reticular chromatin structures, specifically neutrophil extracellular traps (NETs), are important in immune protection, inflammatory diseases, autoimmune disorders, and cancer (80). In OS, characteristics derived from NETs correlate with tumor recurrence and metastasis, and can serve as prognostic indicators for patients (81, 82). Yunhua Lin et al. introduced a novel NETS core that precisely predicts the prognosis of OS patients (81). This score is closely correlated with the immune milieu and treatment response, potentially aiding in guiding clinical decision-making. Currently, research on TANs in OS is still in its early stages, and there is a long way to go in developing anti-tumor treatment strategies related to TANs.

#### 3.1.8 Mast cells

Mast cells constituted a predominant component of the tumor-infiltrating immune cell populations in OS, consistently ranking among the top five most abundant subsets. Immunohistochemical analysis revealed a marked elevation in activated mast cell density within the OS microenvironment compared to non-neoplastic bone tissues (76, 83). Mast cells characterized by dual expression of CD117 and tryptase exhibited predominant localization at the osteolytic interface between tumor and adjacent normal tissues. *In vitro* co-culture studies demonstrated that OS cells actively sustain mast cell viability and functional activation through paracrine signaling (19). T Pathological bone remodeling processes, involving concurrent osteoclastic resorption and disorganized neo-osteogenesis, create chemotactic gradients that enhance infiltration of immunosuppressive myeloid populations into the tumor niche. This dynamic microenvironment paradoxically establishes a feedforward loop facilitating tumor immune evasion. Based on these observations, Inagaki et al. mechanistically linked peritumoral mast cell accumulation with enhanced proteolytic activity via MMP-9 secretion, directly promoting both osteolytic destruction and invasive front progression (84). Importantly, mast cell density at tumor-bone junctions showed strong correlation with radiographic osteolysis severity, validating their utility as a quantitative biomarker for bone destruction monitoring (84).

#### 3.1.9 Immune checkpoints on immune cells

Immune checkpoint molecules are expressed on a variety of immune cells and can be aberrantly activated within the TME, leading to immune suppression. This plays a multifaceted and crucial role in the development, progression, immune evasion, and treatment of OS. A study has demonstrated that the expression of PD-L1 in OS is heterogeneous, with metastatic OS exhibiting higher levels of PD-L1 expression compared to primary tumors (57, 85). CTLA-4 is involved in the negative regulation of T-cell activation and proliferation, thereby suppressing antitumor responses (86). Research has suggested that genetic polymorphisms in CTLA-4 may be associated with the risk of OS development (60, 87). Both TIM-3 and its ligand Gal-9 are expressed in OS, and their interaction promotes T-cell apoptosis within the TME, which is associated with poor prognosis in patients with OS (70). LAG-3 and IDO1 are significantly expressed in pulmonary metastases of OS and are closely related to immune suppression (41). HHLA2, a newly defined member of the B7 family, is widely expressed in OS and is associated with metastasis and poor survival (88). TIGIT is also highly expressed in OS lesions, and blocking TIGIT signaling significantly enhances the cytotoxic activity of T cells, suggesting that targeting TIGIT may have potential therapeutic value in the treatment of OS. Currently, traditional immune checkpoints such as PD-1, PD-L2, and CTLA-4 have been extensively studied, but emerging immune checkpoint molecules hold great potential and warrant further exploration in OS research.

### 3.2 Tumor-suppressing immune cells

The functional dynamics of immune cells within TME transcend simplistic binary categorization as pro-tumorigenic or anti-tumorigenic effectors. Emerging single-cell spatial transcriptomic analyses reveal that these cells exhibit context-dependent plasticity-their phenotypic polarization and functional output are continuously reshaped through intricate crosstalk with malignant cells, stromal components, and metabolic gradients.

#### 3.2.1 TAMs

A high proportion of M1 TAMs is associated with a favorable prognosis in OS (89, 90). This is consistent with the pronounced anti-tumor effects of M1 TAMs (91). A study has demonstrated that a phenotypic shift from M2 to M1 macrophages can lead to regression of pulmonary metastases in OS (92). This aligns with the anti-tumor activity of M1 TAMs, which is related to the production of cytokines that inhibit OS growth (46).Notably, compared to cancers such as gastric cancer or lung adenocarcinoma, high infiltration of CD163^+^ M2-polarized macrophages demonstrates a paradoxical suppressive effect on OS progression through extracellular matrix remodeling-mediated tumor containment (93). Mechanistic investigations by Manara et al. (94) uncovered CD99 as a novel immunomodulatory target that potentiates macrophage-mediated tumoricidal activity. Targeting CD99 on OS cells induces a dual immunogenic switch: (1) inhibitory signal suppression: Downregulation of the “don’t eat me” immune checkpoint molecule CD47 (ligand for SIRPα on macrophages); (2) pro-phagocytic signal activation: Upregulation of “eat me” signals including surface-exposed phosphatidylserine and endoplasmic reticulum-translocated calreticulin (94). In OS, CD47, acting as a transmembrane protein, inhibits phagocytosis by macrophages when it binds to SIRPα on these cells (95).

#### 3.2.2 CD8^+^ T cells

The spatial dynamics and functional heterogeneity of CD8^+^ cytotoxic T lymphocytes (CTLs) constitute pivotal determinants in OS immunobiology. While CTLs are canonically recognized as principal executors of tumor cell elimination through perforin/granzyme-mediated cytolysis, emerging spatial transcriptomic analyses reveal their prognostic value is contingent upon specific intratumoral positioning and metabolic fitness (96, 97). The presence of CD8^+^ T cells in OS is linked to an improved survival rate. A high ratio of CD8^+^ T cells to regulatory (Foxp3^+^) T lymphocytes emerges as a positive prognostic factor for OS patients (98). In OS, the expression of CXCR3 is directly linked to immune infiltration (89). Elevated CXCR3 expression in OS samples, which regulates Ras/ERK, Src, and PI3K signaling pathways, correlates with the activation of CD8^+^ T cells, M1 macrophages, NK cells, plasma cells, monocytes, Tregs, and mast cells (89). A study has shown that CD8^+^ T cells are positively correlated with prognosis (48). As the primary killers of tumor cells in the microenvironment, T cells have become the foremost focus of research in developing immunotherapy.

#### 3.2.3 CD4^+^ T cells

The immunoregulatory role of CD4^+^ T cell subsets in OS pathogenesis has been progressively unraveled through recent advancements in spatial immunophenotyping (99). The absence of CD4^+^ Th1 cells is positively correlated with a high mortality rate in OS (90). CD4^+^ T cells could potentially serve as a prognostic biomarker, and the infiltration of Th1 cells may be correlated with favorable prognosis in OS (90). It has been confirmed that CD4^+^ T cells can directly kill tumor cells via the cytolytic mechanism, and they can also indirectly exert their effect by modulating the TME (100). Furthermore, M1 macrophages can amplify the Th1 cell response by recruiting a significant number of Th17 lymphocytes, establishing a positive feedback loop in the antitumor response (100, 101). 

#### 3.2.4 DCs

DCs, as professional APCs, play a pivotal role orchestrating innate and adaptive immunity. DCs are capable of capturing and processing tumor antigens and presenting them to helper T cells and cytotoxic T cells, thereby initiating anti-tumor immune responses. However, as tumors progress, OS cells evolve immune-evasive variants that can evade the effects of DCs and phagocytic cells. This immunoediting results in diminished DC activation, ultimately allowing tumors to escape immune surveillance (65). A study revealed that in the high immune score group, the number of resting DCs was significantly higher compared to the low immune score group, and the activation level of DCs positively correlated with the response to antitumor therapy (46). Another study examining the immune classification of OS also indicated a negative correlation between DCs and prognosis. CD11c^+^ DCs have been utilized as a source for vaccine immunotherapy, demonstrating positive immune and clinical outcomes (102). Consequently, infiltrating DCs in OS lesions could emerge as future targets for immunotherapy. Currently, vaccination is the most popular treatment strategy for DCs. In OS research, DC vaccines have been tested in preclinical studies and demonstrated the ability to induce tumor immune suppression (103).

#### 3.2.5 B cells

B cells are not only the main players in antibody-producing humoral immunity, but also antigen-presenting cells involved in immune regulation. Lan Li et al. found that CD20 on CD24^+^CD27^+^ B cells and CD20 on IgD^+^CD38^+^ B cells have a negative impact on OS (54). OS patients with highly infiltrated B cells tend to have a better prognosis, and activated B cells are positively correlated with survival rate (104). B cells can actively modulate the tumor immune process by producing anti-tumor antibodies, secreting various cytokines, and acting as antigen-presenting cells. They can also negatively modulate the tumor immune process by inhibiting the proliferation of activated T cells (105). Furthermore, studies indicate that eliminating B lymphocytes not only helps to suppress tumor progression and recurrence but also significantly enhances patients’ sensitivity to chemotherapy (106).

## 4 Strategies and prospects of immunotherapy utilizing immune cells

**Figure 3:**
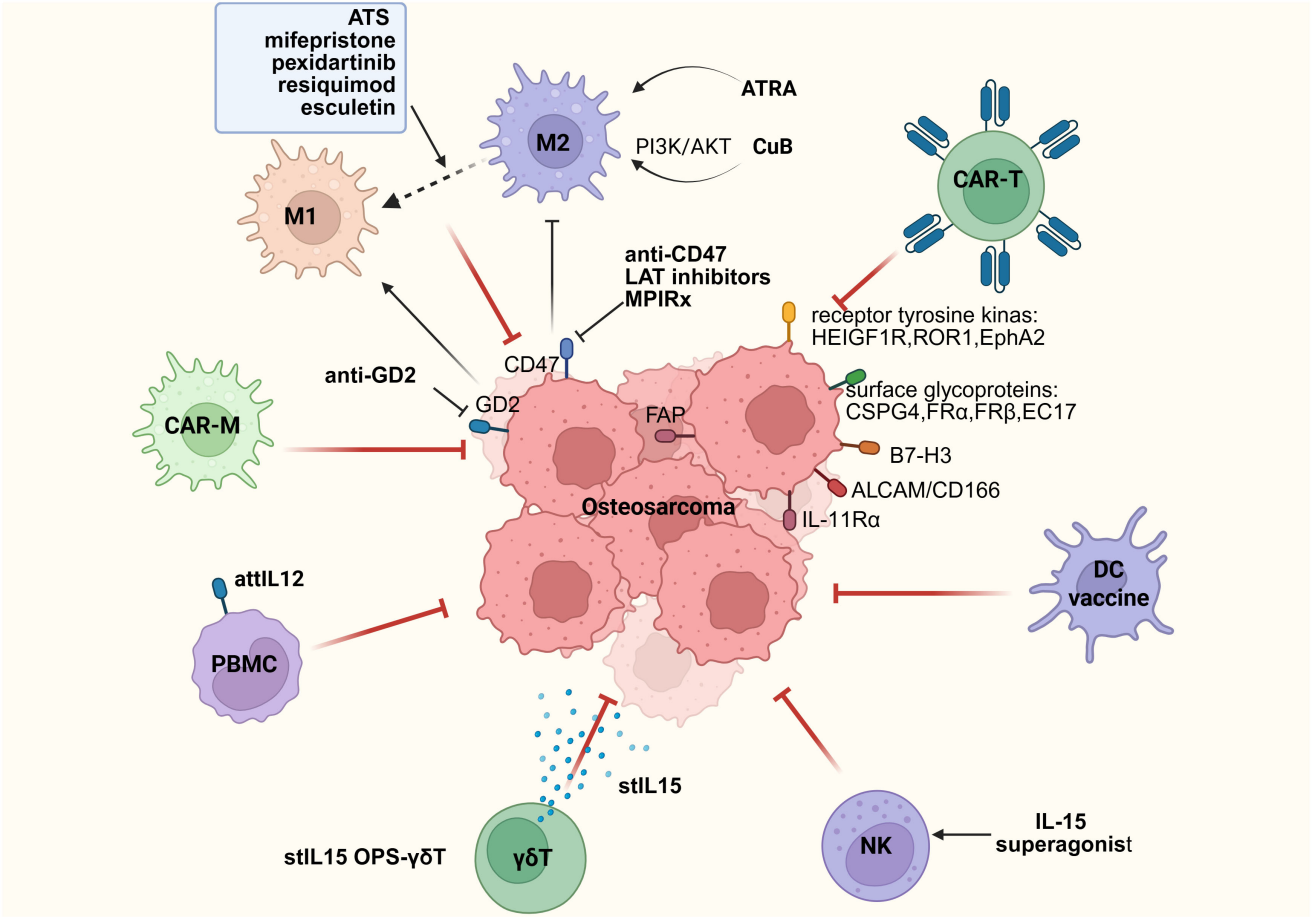
Immune therapy strategies based on immune cells.

Since the introduction of chemotherapy for the treatment of OS in the late 1970s, patients diagnosed with OS have undergone neoadjuvant therapy, followed by postoperative adjuvant therapy. This includes mixed chemotherapy, specifically high-dose methotrexate (12 g/m2), etoposide, and ifosfamide for children and young adults (<25 years old) in the French OS2006/SARCOME-09 study (107), or other regimens combining doxorubicin, cisplatin, and ifosfamide, with or without high-dose methotrexate (108–110). Through these treatment regimens, the 5-year survival rate for children and young adults with localized disease has reached 78%. However, for patients with metastasis at diagnosis or recurrence, the 5-year survival rate remains at only 20% (21, 107). Additionally, over the past 40 years, there has been no significant improvement in the survival rate of patients without metastasis, and no improvement at all for patients with metastasis (15). Therefore, improving the treatment of OS remains a continuous and major goal for many global research and clinical groups. Alannah Smrke et al. proposed the potential role of immunotherapy in treating OS patients (111). The therapeutic target of TME may be key to enhancing the therapeutic effect of OS (**Figure 3**).

### 4.1 Macrophage regulation strategies: polarization reprogramming, immune activation, and CAR-M therapy

The regulation of macrophages within the TME offers a significant opportunity to induce long-lasting antitumor effects. Despite the conflicting roles of TAMs in the TME, three therapeutic strategies targeting TAMs have demonstrated potential in treating OS. (1) Preventing M1 macrophages from polarizing to M2, or directly inhibiting the M2 phenotype. Drug therapies for OS, ATRA (112), resveratrol (113), and dihydroxycoumarin (114), have shown promising results in inhibiting M2-polarized macrophages; (2) Activation of macrophages; (3) Adoptive transfer of CAR-M.

Treating OS by influencing macrophage polarization.

Macrophages serve as crucial mediators in anti-tumor immunity. However, tumors counteract macrophage phagocytosis by expressing the checkpoint molecule CD47, which acts as a “don’t eat me” signal. Studies on both *in vitro* and *in vivo* OS models indicate that anti-CD47 monoclonal antibodies can hinder the CD47-SIRPα signaling pathway, thereby bolstering the anti-tumor capabilities of macrophages (115). Through the use of an anti-GD2/anti-CD47 combination in treating orthotopic OS mouse models, it was discovered that this combined therapy was linked to an increase in macrophage infiltration and the expression of iNOS while reducing the percentage of immunosuppressive M2 macrophages. Furthermore, assessments revealed that anti-GD2/anti-CD47 treatment in neuroblastoma model mice led to an elevation in macrophage count, enhanced iNOS expression, and a decrease in the M2 phenotype TAMs. Research indicated that anti-GD2/anti-CD47 treatment engages these TAMs in the anti-tumor response, facilitating complete tumor eradication (116). Inhibiting L-amino acid transporter 2 (LAT2) or treating tumor cells with LAT inhibitors can downregulate the expression of CD47, enhance macrophage infiltration and phagocytosis of tumor cells, and improve cancer treatment by interfering with LAT2-mediated amino acid uptake (117). The majority of mortality from pediatric OS is caused by lung metastasis (118). In the study conducted by Johanna Theruvath et al. (116), based on a lung metastatic OS model, the combination of anti-GD2 and anti-CD47 nearly eliminated all metastases (116). This approach may represent a method to prevent lung recurrence of OS, an area that urgently requires clinical treatment (116).Natalia Todosenko et al. reported that a high proportion of M1 macrophages is associated with a favorable prognosis in OS, aligning with the pronounced antitumor effect of M1 macrophages. The shift from M2 to M1 phenotype has been demonstrated to facilitate the regression of lung metastatic OS, with its antitumor potency linked to the production of cytokines that suppress OS growth (93). In research targeting lung metastasis of OS, MPIRx (a biomimetic nanodrug composed of a sonosensitizer and a CD47 inhibitor) is capable of directing macrophages towards tumor cells, fostering M1 polarization, and enhancing the phagocytic activity of macrophages towards OS cells (119).

Inhibiting or blocking M2 polarization for OS treatment. ATRA prevents the migration of OS cells both *in vitro* and *in vivo* by suppressing M2 polarization induced by interleukin IL-13 or IL-14 (112). ATRA can diminish the cancer stem cell (CSC) traits bolstered by the M2 phenotype, including the rise in the number of CD117^+^Stro-1^+^cells and the overexpressed CD133, CXCR4, Nanog, and Oct4 (45). Research has shown that cucurbitacin B (CuB) can also hinder the differentiation of M2 macrophages via the PI3K/AKT pathway, thus impeding OS progression (120). A decade ago, studies reported that in a mouse model implanted with human OS, tumor growth was slowed by eliminating TAMs. Furthermore, when the epidermal growth factor receptor is suppressed, tumor growth stimulated by recruited and polarized macrophages is inhibited (121). TAM-specific surface molecules represent ideal targets for drug development. Pexidartinib (PLX3397), a colony-stimulating factor 1 receptor inhibitor, holds the potential to reprogram TAMs and activate T cell infiltration into OS, ultimately reducing tumor growth and lung metastasis (122). Additionally, analogous drugs include terpenoids asiaticoside (ATS), mifepristone, pexidartinib, resiquimod, esculetin, among others (123, 124). Recent research has revealed that copper and magnesium ions can also treat OS through macrophage polarization (125, 126).

Macrophage Activators

Some macrophage activators can also be utilized for the treatment of OS. Selectively inhibiting immunosuppressive cells (MDSCs and TMAs) in the microenvironment *in vitro* results in the reprogramming of the TME, making it more favorable for tumor immunity (127). Furthermore, studies have shown a significant improvement in the survival rate of patients who develop deep infections within 12 months following surgery (128). Immune activation linked to infection likely contributes to enhancing the prognosis of these patients. In certain countries and regions, mifamurtide, a macrophage activator, has been approved for the treatment of OS, potentially improving patient prognosis (111). L-MTP-PE (liposomal muramyl tripeptide phosphatidylethanolamine; Mifepristone) is a synthetic drug that stimulates immune responses and activates macrophages and monocytes (129). It is believed that L-MTP-PE influences the progression of high-grade OS by affecting the M1/M2 polarization of TAMs (130), and provides evidence of controllable safety (131). Nanomaterial delivery of chemotherapeutic agents and immunomodulators can also activate macrophages, thereby enhancing antitumor effects (132). Additionally, TAMs can be activated by targeting CD47, hence anti-CD47 monoclonal antibodies have been proposed as anticancer drugs, aiming to reactivate TAMs in the TME setting (133).

Adoptive transfer of CAR-M

Macrophages, as the most prevalent immune cell component infiltrating the OS microenvironment, hold significant potential as a tool for adoptive immunotherapy. Currently, macrophages have been engineered to express chimeric antigen receptors (CARs) targeting cancer-specific antigens, known as CAR-M cells. Research on CAR-M is primarily in its infancy. Michael Klichinsky et al. (134) discovered that CAR-Ms exhibited antigen-specific phagocytosis and tumor clearance capabilities *in vitro*. In humanized mouse models, CAR-Ms can further induce a pro-inflammatory TME and enhance anti-tumor T cell activity (134). Additionally, CAR-Ms promote an inflammatory state within the TME and are capable of cross-presenting antigens to tumor-specific CD8^+^ T cells (135).

In OS, CAR-M stands as a promising tool capable of specifically recognizing and eliminating tumor cells (70). Research indicates that, compared to CAR-T cells, CAR-M cells possess advantages in infiltrating the TME, circumventing the immunosuppressive milieu, and re-educating “bystander” M2 macrophages. The inaugural human CAR-M trial has commenced, targeting HER2-positive solid tumors (clinical trial NCT04660929) (136). Engineered CAR-Ms have led to an elevation of anti-tumor cytokines (like IL-6) and chemokines (such as CXCL18) within the TME of OS. The generation of these advantageous cytokines aids in converting cold tumors into hot tumors (70). Ultimately, TAMs emerge as potential targets for innovative therapeutic approaches.

### 4.2 CAR-T cell therapy and synergistic regulation of the immune microenvironment

Adoptive T cell transfer is currently one of the research hotspots, involving the introduction of specific T cells expanded *in vitro* into patients to supplement and enhance T cell-related immunity. Common applicable T cells include CTLs, γδ T cells, and genetically engineered tumor-specific T cells (137). Chimeric antigen receptor T cells (CAR-T) exhibit significant efficacy in liquid tumors, yet show limited response in solid tumors. To ensure the effectiveness of CAR-T cell therapy, two primary criteria must be met: (1) CAR-T cells should target epitopes selectively expressed on the surface of OS cells, thus avoiding toxicity to normal tissues; (2) this target should also be widely expressed in OS metastases (138). Lucía Fernández et al. (139) evaluated the safety and cytotoxic activity of CD45RA-memory T cells expressing the NKG2D-4-1BB-CD3ζ CAR against OS cells. They discovered that by enhancing the interaction between NKG2D ligands and receptors in OS, the antitumor activity of NKG2D-CAR memory T cells was significantly boosted (139). Sabina Kaczanowska et al. conducted a phase I trial (NCT02107963) of GD2-CAR-T, demonstrating the feasibility and safety of its administration in children and young adults suffering from OS and neuroblastoma (140). Most potential targets for CAR-T cell therapy in OS are tumor-associated antigens. Some current potential targets for CAR-T cell therapy in OS include receptor tyrosine kinases, cell surface glycoproteins, B7-H3 (CD276), disialoglycoside (GD2), NKG2D, activated leukocyte adhesion molecule, interleukin-11 receptor α (IL-11Rα), and fibroblast activating protein (141).

These data collectively indicate that CAR-T can be utilized to advance immunotherapy for patients with solid tumors. However, merely enhancing the adaptability of effector T cells to OS is insufficient. It is also crucial to promote their proliferation, prolong their lifespan, strengthen their resistance to the inhibitory immune microenvironment, and increase their susceptibility to tumor cells. GD2-CAR-T cells exhibit good safety, yet their antitumor response is limited. Kaczanowska et al. found that at baseline, the proportion of CXCR3^+^ monocytes in apheresis blood components and peripheral blood was significantly higher among patients with good expansion, suggesting that CXCR3^+^ monocytes may affect the function of CAR-T cells (142). Recent studies have reported the potential of CXCR5 and IL-7 co-expression in enhancing the efficacy of CAR-T cell therapy for OS (143). CAR-T cell therapy has demonstrated effectiveness against OS in preclinical studies, and promising results have also been observed in clinical trials targeting specific antigens. However, challenges such as low antigen specificity, limited durability, significant side effects, and an unfavorable TME have hindered the wider application of CAR-T therapy in OS. To overcome these limitations, future advancements may include the development of novel CAR-T cells, combination drug therapies, and gene editing techniques, aiming to enhance the efficacy and safety of CAR-T cell therapy in treating OS.

In addition, there are non-cellular therapies designed based on the characteristics of T-cell infiltration in the immune microenvironment. In OS models, the L19-tumor necrosis factor alpha fusion protein can selectively target tumor vessels and synergistically enhance the antitumor effects of melphalan and gemcitabine (144). Furthermore, there was an increase in CD4^+^ and CD8^+^ T cells within tumor infiltrates, while Tregs were significantly reduced in MDSCs and draining lymph nodes (144). Chiara Ratti et al. found that in immunogenic OS models, trabectedine increased the number of tumor-infiltrating T lymphocytes. However, due to the high expression of the inhibitory checkpoint molecule PD-1, local CD8^+^ T cells may be in the late stage of activation or exhaustion. Therefore, combining trabectedine with a PD-1 blocking antibody significantly improved its efficacy in controlling OS progression (145). Coincidentally, TIGIT blockade enhanced the cytotoxic effect of primary CD3^+^ T cells with a high proportion of TIGIT^+^ cells on OS (42).

### 4.3 DCs as antigen-presenting engines: multimodal vaccine strategies, immune microenvironment remodeling, and combination therapies

DCs, as professional APCs, absorb and present antigens to naive T cells, ultimately prompting their differentiation into tumor-killing cells. Recent studies indicate that DCs can also activate γδ T cells, cytokine-induced killer cells, and other natural immune cells that exhibit potent anti-tumor activity. The primary mechanism behind DC therapy is to restore antitumor immunity in the body. Nevertheless, established tumors consistently endeavor to diminish the availability of antigen presentation by APCs, resulting in immunosuppression and subsequently impeding the initiation of anti-tumor immune responses. Vaccination stands as the most prevalent treatment for DCs.

DC vaccines have been developed to circumvent this mechanism. The process can be summarized as follows: DCs are isolated from peripheral blood mononuclear cells (PBMCs), matured, and loaded with tumor antigens *in vitro*, before being injected into patients. Theoretically, these antigen-activated DCs can effectively bolster the immune response. Based on the methods of loading different antigen sources, they can be categorized into three main types: (1) DCs co-cultured with peptides, proteins, or tumor cell lysates; (2) DCs transfected with DNA, RNA encoding antigens, or total RNA of tumor cells; (3) DCs fused with inactivated tumor cells. Yu et al. tested the efficacy of OS DC vaccines fused with whole tumor cells or transduced with total tumor RNA. Most of the immunized tumor-free rats achieved partial or complete protection against tumor challenge. Additionally, vaccination was found to induce tumor suppression in tumor-bearing mice (146, 147). Other studies explored the potential of combination therapy involving DC vaccines and targeted drugs, such as anti- TGF-β/glucocorticoid-induced tumor necrosis factor receptor antibodies (148). These findings indicated that both primary and metastatic tumor growth were inhibited. Furthermore, the TME was reshaped, with a decrease in the number of Tregs, reduced levels of immunosuppressive cytokines, and an increase in the number of CD8^+^ T cells (148).

Several vaccines have demonstrated encouraging efficacy, including the CD11c^+^ DC vaccine and polyinosinic acid vaccine. Specifically, poly I:C activates and loads tumor antigens onto CD103^+^ myeloid/conventional DC1 cells. The CD103^+^ cDC1 vaccine, generated *in vitro*, can induce systemic and long-lasting tumor-specific T cell-mediated cytotoxicity, ultimately suppressing the growth of both primary and metastatic tumors (149). The study revealed that treating with K7M3 OS cell lysate-derived CD103^+^ DC vaccine suppressed tumor growth and boosted the number of T cells in these tumors and lymph nodes. Combined therapy with anti-CTLA-4 and DC vaccines enhances the effectiveness of DC vaccines against lung metastases (150). Multi-antigen stimulated cell therapy, which involves loading DC vaccines with multiple antigens, followed by adoptive transfer of anti-tumor effector T cells, has been shown to be safe and effective in treating advanced bone and soft tissue sarcomas when combined with cabozumab and apatinib (151). Nevertheless, DC vaccines have demonstrated limited efficacy in clinical trials for OS treatment (152, 153). For instance, among 12 patients, only 2 exhibited a robust anti-tumor immune response following a 3-week DC vaccination regimen, and none demonstrated any clinical benefit (154). Three explanations can be proposed for the lack of clinical benefit for patients: (1) The quality and quantity of immune effector cells in patients are compromised. Patients with OS typically undergo a full course of neoadjuvant chemotherapy, which may impair both innate and adaptive immune responses, thereby limiting their availability and effectiveness in dealing with increased antigen presentation; (2) The poor migration ability of effector cells to the tumor site may be attributed to the downregulation of chemokine expression; (3) Other potent immunosuppressive mechanisms, such as immune checkpoints on immune cells. An effective cancer vaccine should be able to overcome tumor-associated immunosuppression and restore immune surveillance (155). Therefore, increasing the ratio of active effector cells to tumor target cells, enhancing the infiltration of effector cells, or remodeling the TME in combination with DC vaccines can enhance antigen presentation, immune response, and clinical efficacy.

In addition to vaccines, liposomal muramyl tripeptide phosphatidylethanolamine, whether used alone or in combination with other methods, has the potential to prolong overall survival and metastasis-free survival by activating DCs or generating T cells (156). Kawano et al. combined DCs with anti-TGF-β antibodies to treat OS, detecting an enhanced systemic immune response *in vivo (* 157). Previous attempts have focused on utilizing DCs to maximize tumor killing by enhancing lymphocyte immune activity. However, these studies also highlighted the tumor-promoting activity of DCs, posing a significant risk in DC-related therapies. Existing research has unveiled the therapeutic potential of DCs, with certain drugs or drug components amplifying their effects. For instance, capsaicin has been reported to enhance the phagocytosis of OS cells by DCs *in vitro (* 158). Leveraging their strengths while mitigating their weaknesses, and pursuing more precise treatments, are crucial for the future application of DCs.

### 4.4 NK cell-driven OS immunotherapy: innovations in multimodal therapies, synergistic strategies, and pathways to overcome translational medicine bottlenecks

Tumor cells evade adaptive immune surveillance through antigen shedding, downregulation of major histocompatibility complex I, and suppression of T cells. The use of NK cells, either alone or in combination, demonstrates significant potential in this regard. NK cell therapy for OS primarily revolves around three approaches: adoptive NK cell therapy, cytokine-based targeted therapies to enhance NK cell immune activity, and chimeric antigen receptor NK cells (CAR-NK). Childhood sarcoma cells have been proven to be highly sensitive to NK cell-mediated killing. NK cell adoptive therapy offers numerous advantages over T cell adoptive therapy, including excellent safety and the absence of major histocompatibility complex limitations. NK cell immunotherapy holds the promise of becoming a novel treatment for malignant bone tumors in children (159). Adoptive NK cells have achieved initial success in the treatment of OS (160). The rationale behind this is to reactivate suppressed NK antitumor immunity. NK cells used for treatment can be obtained from autologous or exogenous sources such as peripheral blood, umbilical cord blood, hematopoietic progenitor cells, and pluripotent stem cells. The advantages of this approach lie in its safety and the absence of graft-versus-host disease. In a study examining KIR receptor-ligand incompatibility of NK cells against OS cell lines, mismatched allogeneic donors in OS may exhibit greater antitumor effects compared to matched or autologous NK cells (161). IL-15 superagonist and denosumab enhanced the viability and proliferation of peripheral blood NK cells expanded *in vitro*, significantly prolonging the survival time of mice with OS (162). IL-15-induced allogeneic and autologous NK cells increased the sensitivity of chemotherapy-resistant OS cells (163). These two studies underscored the application value of NK cell activator IL-15 in OS. CAR-NK is a novel and promising therapy that loads specific antibodies onto NK cells, and it has been observed to exhibit therapeutic effects on Ewing’s sarcoma and B-cell leukemia (164). Nevertheless, research on CAR-NK cells in OS remains scarce. Up to now, both CAR-NK and CAR-M therapies have not yielded encouraging progress in OS research. It’s worth noting that these two therapies could theoretically compensate for the limitations of CAR-T therapy, yet their clinical application still has a long way to go.

Another crucial approach to enhance the antitumor efficacy of adoptive transfer of NK cells is through combination therapy. Given the ADCC carried out by NK cells, the use of monoclonal antibodies emerges as an obvious option for combination therapy. Nevertheless, one of the challenges posed by immune-based combination therapy is the potential risk of immune-related adverse reactions. The combination of autologous NK cells and anti-PD-L1 monoclonal antibody has demonstrated good tolerability in heavily pretreated patients with advanced sarcoma (NCT03941262). CIML NK cells exhibit enhanced ADCC capabilities. A clinical trial is currently assessing the safety and effectiveness of CIML NK cells, in conjunction with an IL-15 super antagonist and ipilimumab, for the treatment of advanced head and neck cancer (NCT04290546). Other potential monoclonal antibodies for combination with NK cell ACT include those targeting inhibitory NK receptors, such as anti-NKG2A (e.g., monalizumab) and anti-KIR2D (e.g., lililumab), as well as antibodies targeting TAAs, like anti-IGF1-R (e.g., cixutumumab) (159).

### 4.5 γδT cell engineering therapy: bystander immune synergy

γδ T cells are an emerging alternative for cell therapy, featuring innate antitumor activity, potent antibody-dependent cytotoxicity, and minimal alloreactivity. Recently, Daniel Fowler et al. (165) proposed an immunotherapy platform technology leveraging the inherent characteristics of Vγ9Vδ2 T cells. This technology capitalizes on the specific traits of this cell type, offering a fully compatible cell therapy capable of eliciting bystander immunity (165). They engineered γδ T cells to synthesize and secrete tumor-targeting opsonins and a mitogenic IL-15Rα-IL-15 fusion protein (stIL15). Their data indicated that Vγ9Vδ2 T cells secreting GD2-specific opsonin (stIL15-OPS-γδ T cells) exhibited enhanced cytotoxicity and were capable of promoting the bystander activity of other lymphoid and myeloid cells, thereby mediating the activation of bystander NK cells (165). These stIL15-OPS-γδ T cells have demonstrated therapeutic efficacy in patient-derived OS, and this efficacy can be further enhanced by the addition of zoledronic acid (165). This suggests that modified γδ T cells emerge as a promising allogeneic cell therapy platform, integrating direct cell lysis with bystander activation to facilitate tumor control.

### 4.6 Others: novel PBMC cell engineering and combined immunotherapy

CAR-T cell therapy demonstrates significant potential in treating hematological malignancies, yet it necessitates prolonged T cell expansion, incurs severe toxicity, and exhibits limited effectiveness in solid tumor treatment. Consequently, Qing Yang and colleagues devised an anchored cell membrane and tumor-targeting IL12 (attIL12) to equip PBMCs. This IL12-based attIL12 PBMC therapy demonstrated remarkable antitumor efficacy in both xenograft tumors derived from patients with heterogeneous OS and metastatic OS, without evident toxic effects (166). In addition, combined immunotherapy is increasingly being explored in the treatment of OS. Ocadlikova et al. conducted *in vitro* studies demonstrating that treatment of cell lines with the tyrosine kinase inhibitor sunitinib led to increased PD-L1 expression and facilitated the activation of immune cells (167), bolstering the case for combined immunotherapy strategies. Furthermore, the suboptimal outcomes of single-agent immunotherapy in clinical trials might be attributed to the immunosuppressive effects of the TME. Recent *in vitro* research has shown how selectively inhibiting immunosuppressive cells (MDSCs and TAMs) within the microenvironment can reprogram it towards a more immune-friendly state (127). Potential future research avenues might involve integrating immunotherapy with drugs that enhance the immune-activating microenvironment, particularly for patients exhibiting low or average immune infiltration characteristics, employing a precision medicine-inspired approach.

### 4.7 Immune checkpoint inhibitors

The SARC028 trial (NCT02301039) was the first study to evaluate the activity and safety of anti-PD-1 antibodies in patients with soft tissue sarcoma and OS, with a response rate of 5% and acceptable adverse events (168). Another single-arm, open-label Phase II trial (NCT03013127) demonstrated that pembrolizumab was well-tolerated but did not show clinical benefit (169). Additionally, a Phase II study (NCT02406781) confirmed that the combination of pembrolizumab and cyclophosphamide resulted in only one patient achieving partial response, and expression levels of PD-L1 were not directly correlated with antitumor efficacy (170). A Phase I clinical trial showed that ipilimumab (an anti-CTLA-4 antibody) had excellent safety, increased the number of activated and proliferating T cells, but did not increase the number of regulatory T cells (171). In OS models, blocking TIM-3 inhibits tumor growth, increases the number of tumor-infiltrating CD8^+^ T cells, and activates their function (62). To date, clinical trial results for TIM-3 inhibitors in OS have not been published. However, TIM-3 remains an important potential target for future research. Current research findings indicate that immune checkpoint inhibitors have not shown satisfactory antitumor effects in OS. A deeper investigation into the underlying mechanisms of immunotherapy may provide new directions and insights for its application in OS.

The above studies collectively demonstrate that in the field of OS treatment, preclinical research on macrophages, CAR-T therapy, and other immunotherapies has shown significant translational potential, while also facing numerous challenges. From a translational application perspective, several innovative pathways have emerged. In terms of macrophage reprogramming and targeted regulation, anti-CD47 combination therapies enhance macrophage phagocytic activity by blocking the CD47-SIRPα signaling pathway (115). Combined with anti-GD2 antibodies, these therapies can nearly eradicate pulmonary metastases in preclinical models (116). Additionally, MPIRx nanoparticles have been shown to inhibit the growth of metastatic OS in mouse models (119). CAR-M cells exhibit antigen-specific phagocytic capabilities and T-cell activation in both *in vitro* and *in vivo* models, and can bypass immunosuppressive effects in the TME when combined with gene-editing technologies (134, 136). CAR-T therapy has also made progress through optimized target selection and engineering design. For instance, GD2-CAR-T therapy has demonstrated good safety in pediatric OS patients. Novel targets and co-expression designs can enhance T-cell function, and combining TME modulators or checkpoint inhibitors may help overcome barriers in solid tumors. γδ T cells and other allogeneic universal platforms, which do not carry the risk of graft-versus-host disease, are suitable for the development of “off-the-shelf” treatments. Furthermore, DC vaccines combined with anti-CTLA-4 can inhibit pulmonary metastases in preclinical settings, and future integration of neoantigen prediction technologies may improve antigen specificity. NK cell adoptive therapy and CAR-NK cells also show potential in OS treatment by extending survival and enhancing antibody-dependent cellular cytotoxicity.

However, current research still faces many challenges. Preclinical models have limitations, such as the inability of mouse models to fully simulate the heterogeneity of patient lesions and immune damage caused by pre-treatment with chemotherapy. The complexity of the TME is also a significant concern, with high infiltration of various immunosuppressive cells that cannot be fully reversed by existing therapies. Additionally, the heterogeneous antigen expression of OS cells can lead to immune escape. Cell therapies also face issues of persistence and safety, with engineered cells being prone to exhaustion in solid tumors and carrying the risk of off-target toxicity. The preclinical achievements in OS immunotherapy reveal the great potential of macrophage reprogramming, CAR engineering, and combination therapies, but overcoming the challenges of model limitations, TME heterogeneity, and cell persistence is essential for future progress.

## 5 Conclusions and prospects

As the predominant primary bone malignancy in pediatric and adolescent populations, OS continues to pose formidable therapeutic challenges. The current standard of care has improved the prognosis for patients with localized OS, yet the overall survival rate for OS patients has remained relatively unchanged for over 30 years. There is a need to develop more effective treatments for patients with high-risk characteristics, while also minimizing treatment-related toxicity for all patients. Predictive biomarkers are essential to assist clinicians in adjusting treatment, including immunotherapy, and to facilitate the design of future clinical trials. Studies indicate that tumor cells may persistently engage in a battle with the immune system, potentially disrupting the balance at some point. Once a tumor forms, it becomes challenging to eliminate it completely. Immunity holds promise for eliminating tumor cells from the body at the cellular level.

Over the past decades, with the introduction of immunotherapy, OS treatment strategies have undergone development and improvement. However, they remain ineffective and fail to provide a complete response for patients. There are numerous reasons for the failure of immunotherapy in OS, including the presence of TAMs in TME or tumor or metastatic tissues, as well as the infiltration of macrophages, immune cells, and other bone cells. OS is a solid tumor, making it challenging to penetrate the thick fibrous primary tissues, which further impedes immunotherapy. Other factors may also include resistance to treatment and the absence of effective contact between T cells and tumor cells, which hinders T cells from secreting IFN-γ or triggers insufficient signals for cytokines responsible for tumor suppression. Additionally, the absence of immune tumor cells or antigen presentation fails to provide enough antigens for T cells to recognize. Some studies indicate that immunotherapy could potentially cause hepatorenal toxicity and even cardiac arrest in patients. Hence, by targeting specific cell populations within the TME niche, instead of multiple cell types, we can overcome the issue of tumor progression. For instance, the repolarization of M2/TAM macrophages stands as a pivotal field in cancer immunotherapy, broadening the scope for targeted therapies against diverse cell types within the TME. Given the heterogeneity of M2/TAM, a deeper exploration of its repolarization is warranted. Consequently, reshaping or re-educating the target macrophage population emerges as a crucial strategy for therapeutically inhibiting tumor growth and achieving tumor suppression through macrophage-targeted approaches. Furthermore, immune checkpoint inhibitors (ICPIs) signify a cutting-edge advancement in cancer treatment, demonstrating notable therapeutic efficacy in OS patients. Certain immune checkpoints are expressed not only on T cells but also on DCs, macrophages, NK cells, NKT cells, and γδ T cells; blocking these checkpoints can reverse their antitumor activity in tumor immunity. Therefore, immunotherapy is emerging as a potential strategy for the treatment of OS. Based on the current preclinical and clinical data regarding immunotherapy, there is still a long way to go for its application in OS.

Additionally, the response rate of monotherapy in OS is relatively low. Combination therapy based on immunotherapy could be the future direction. The next-generation therapeutic paradigms are anticipated to incorporate multidimensional combinatorial strategies (**Table 1**) and artificial intelligence-powered treatment personalization, as evidenced by recent advances in precision oncology.

**Table 1:** Multidimensional combination strategies.

Component	Mechanistic synergy	Clinical trial
CAR-Macrophages	Phagocytosis+antigen cross-presentation	NCT05520345 (Phase I/II)
Oncolytic virus+anti-CTLA4	Viral immunogenic cell death enhancement	MAESTRO-OS platform
Epigenetic modulators	Demethylation of endogenous retroviral genes	PROSPECT-OS consortium trial
